# Enhanced Photoelectrical Response of Hydrogenated Amorphous Silicon Single-Nanowire Solar Cells by Front-Opening Crescent Design

**DOI:** 10.1186/s11671-016-1447-0

**Published:** 2016-04-29

**Authors:** Zhenhai Yang, Guoyang Cao, Aixue Shang, Dang Yuan Lei, Cheng Zhang, Pingqi Gao, Jichun Ye, Xiaofeng Li

**Affiliations:** College of Physics, Optoelectronics and Energy & Collaborative Innovation Center of Suzhou Nano Science and Technology, Soochow University, Suzhou, 215006 China; Ningbo Institute of Material Technology and Engineering, Chinese Academy of Sciences, Ningbo, 315201 China; Key Lab of Advanced Optical Manufacturing Technologies of Jiangsu Province and Key Lab of Modern Optical Technologies of Education Ministry of China, Soochow University, Suzhou, 215006 China; Department of Applied Physics, The Hong Kong Polytechnic University, Hong Kong, China

**Keywords:** Single-nanowire solar cells, Coupled optoelectronic simulation, Light-trapping, Crescent, 85.60.-q, Optoelectronic device, 84.60.Jt, Photovoltaic conversion

## Abstract

We report an approach for substantially enhancing the light-trapping and photoconversion efficiency of hydrogenated amorphous silicon (a-Si:H) single-nanowire solar cells (SNSCs) by engineering the cross section of the nanowire from circular into a front-opening crescent shape. The proposed SNSCs show a broadband and highly tunable optical absorption compared to the conventional circular counterparts under both transverse electric and transverse magnetic incidences, enabling an enhancement ratio of over 40 % in both the photocurrent density and the photoconversion efficiency in a-Si:H SNSCs with a diameter of 200 nm. We further show that the superior performance can be well maintained under a wide range of incident angle and is robust to the blunt crescent edges.

## Background

Single semiconductor nanowires (NWs) have been explored extensively over the past decades due to their unique nanoscale morphology and outstanding photoelectric response, which can find a variety of potential applications, e.g., photovoltaics (PV) [1–4], photodetectors/sensors [5–7], light-emitting diodes [8–10], and thermoelectric devices [11–13]. Particularly, compact single-nanowire solar cells (SNSCs) have attracted considerable attention due to their excellent light-trapping ability, efficient carrier collection, and convenient integration into chips. However, the potential of SNSCs in realizing highly efficient nanoscale PV devices has not been fully exploited. From the fundamental light-trapping to the intrinsic photoconversion processes, there still exist many challenges. In order to improve the light-trapping performance, coaxial heterojunction [14, 15], dielectric-shell coating [16, 17], rear reflector [18, 19], and metal-core/semiconductor-shell NWs [20, 21] have been proposed. In electrical perspectives, photocurrent matching (for double-junction SNSCs) [15], electrical doping, recombination loss [22], etc. have to be carefully addressed as well.

Among various performance-modulation strategies, optimizing the cross-sectional morphology of NWs is an easy way to enhance the light-harvesting response [23–26]. For instance, Kim et al. showed that the rectangular silicon NWs have enhanced external quantum efficiency (EQE) than their circular counterparts at long wavelengths [23]. Cao et al. proposed a series of SNSCs with various cross sections including circle, square, hexagon, and triangle, which, however, seem to display similar optical response if taking the volume difference into account [24]. We recently found that the light-trapping ability of silicon-based SNSCs can be significantly improved by introducing the symmetry-broken rear-crescent design, benefitted from the dramatically enhanced absorption peaks, especially in the long-wavelength band [26].

In this study, we focus on the application of crescent configuration in improving the light-trapping and photoconversion performance of SNSCs consisted of highly absorbing hydrogenated amorphous silicon (a-Si:H). We consider different crescent orientations against the solar incidence as well as the comparison between the crescent and the conventional circular systems. Finite-element method (FEM) and carrier-transport calculations are employed to predict the optical and electrical performance of the a-Si:H SNSCs, respectively. The absorption patterns under various cavity resonances are used to uncover the physics behind the performance enhancement. It shows that the photocurrent density (*J*_ph_) can be increased from 14.38 to 20.22 mA/cm^2^ by the optimized crescent design when comparing the circular counterpart; moreover, the outstanding light-harvesting capability can be well sustained in a wide range of incident angle or with the presence of blunt crescent tips. Simulation on carrier transportation and collection predicts an enhancement ratio of 43.77 % in light-conversion efficiency over the circular system.

## Methods

**Fig. 1:**
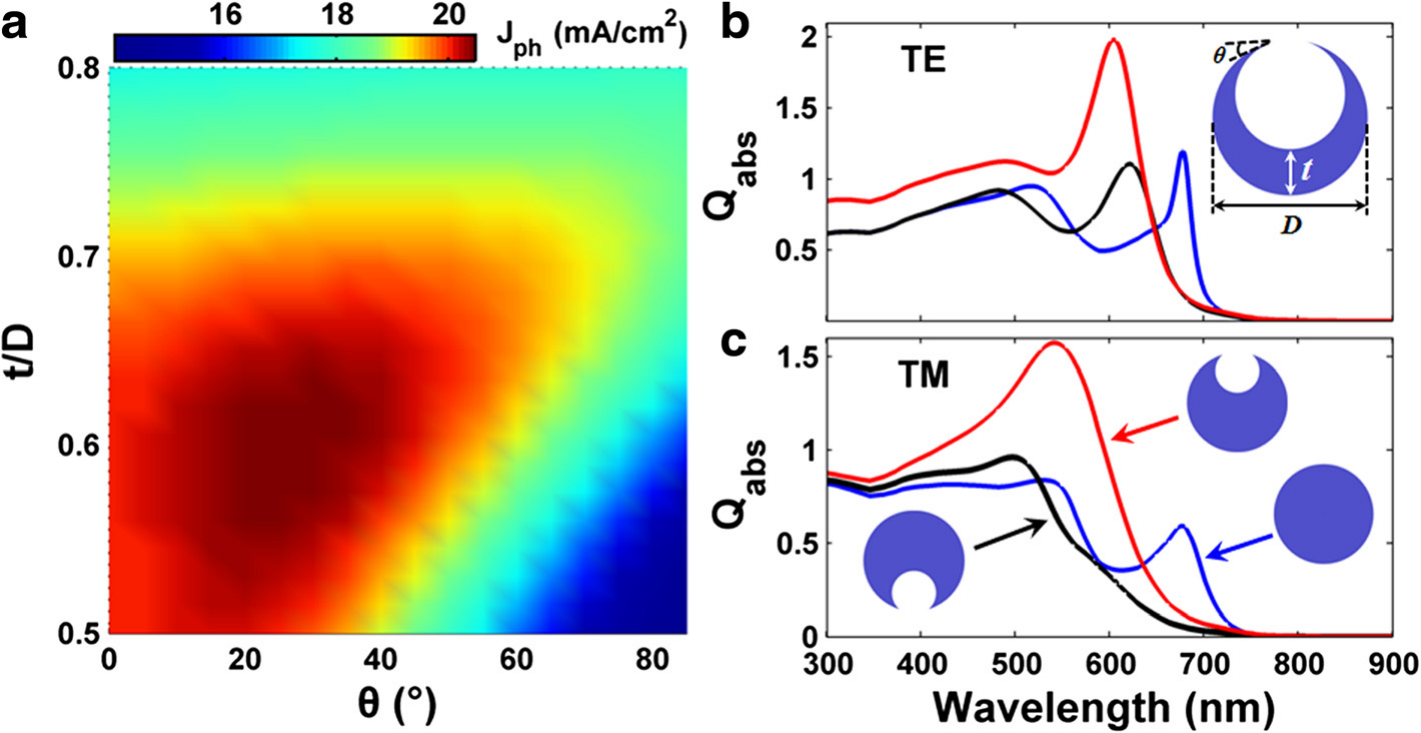
Photocurrent map and absorption spectra. **a** Photocurrent density (*J*_ph_) versus thickness/diameter (*t*/*D*) and vertex angle (*θ*) for the front-opening crescent a-Si:H SNSCs under unpolarized incidence; absorption efficiency (*Q*_abs_) of the circular (*D* = 200 nm), rear- and front-opening crescent (*D* =200 nm, *t*/*D* = 0.6, *θ* = 25°) systems for TE (**b**) and TM (**c**) incidences

The cross-sectional configuration of a-Si:H SNSCs with front-opening crescent design is shown by the inset of Fig. 1b, where the NW diameter *D*, crescent thickness *t*, and vertex angle of the crescent tip *θ* are the key parameters to characterize the device geometry [26, 27]. The length of the lying NW is far beyond the cross-sectional sizes, allowing the two-dimensional (2D) simulation. To predict the performance of SNSCs, transverse electric (TE, electric field along the NW) and transverse magnetic (TM, magnetic field along the NW) incidences have to be considered so that the unpolarized solar incidence can be approximated as the average of TE and TM cases. The optical parameters of a-Si:H are taken from Palik’s data [28]. The absorption percentage of the cell (*Q*_abs_) can be obtained by solving Maxwell’s equations, and an estimated (assuming perfect internal photoconversion progress) photocurrent density (*J*_ph_) is obtained by spectrally integrating *Q*_abs_ weighted by the AM1.5G solar spectrum within the wavelength range of 300~900 nm [29, 30]. The actual electrical response (EQE, short-circuit current density (*J*_sc_), current-voltage characteristics, etc.) of the SNSCs can be achieved by performing the detailed carrier transport/collection calculation, which is based on the carrier drift-diffusion and Poisson’s equations coupled with Maxwell’s equations. It is obvious that both *J*_ph_ and *J*_sc_ subject to unpolarized incidence should be the average of TE and TM cases. The detailed information on the coupled optoelectronic simulation can refer to our previous publications [31–35].

## Results and Discussion

First of all, we focus on how to modulate the optical response of the a-Si:H SNSCs (*D* = 200 nm) by controlling the crescent parameters *t* and *θ*. Fig. 1a plots *J*_ph_ as a function of the tunable parameters *t* (100 nm < *t <* 160 nm) and *θ* (0° < *θ* < 85°), under an unpolarized incidence. Apparently, the value of *J*_ph_ shows strong dependences on both *t* and *θ*, showing that the cavity and crescent factors play very crucial roles in determining the absorption performance of the considered SNSCs. With an optimal crescent design, i.e., *t*/*D* = 0.6 and *θ* = 25°, peaked *J*_ph_ exceeding 20 mA/cm^2^ can be realized. Particularly, Fig. 1a also shows that there are quite wide ranges for *t* and *θ* to obtain a *J*_ph_, which can be much higher than that of the conventional circular-shaped SNSCs.

Figure 1b (Fig. 1c) provides a detailed comparison of the absorption responses of the SNSCs with circular and front- and rear-opening crescent configurations under TE (TM) incidence. We first compare the rear-opening crescent and circular systems. Figure 1b indicates that under TE incidence, *Q*_abs_ shows an unnoticeable change in short-wavelength (*λ* < 450 nm) range but a strong oscillation in long-wavelength range when comparing to the circular one. Under TM incidence, however, performance enhancement (degradation) is observed when *λ* < (>) 520 nm, as displayed in Fig. 1c. As a whole, under TE (TM) incidence, the *J*_ph_ of rear-opening crescent cells is only 14.89 (11.01) mA/cm^2^, which is even lower than that based on the traditional circular system, i.e., 15.35 (13.41) mA/cm^2^. Attention should be paid here on that SNSCs composed by low-absorbing crystalline silicon (c-Si) show dramatically improved *J*_ph_ with the rear-opening crescent design, due to the significantly enhanced cavity resonance [25, 26]. However, for the a-Si:H SNSCs which consisted of a much smaller cavity, the supported cavity modes are much less in number, so that the rear-opening crescent design can hardly play a similar effect in enhancing the absorption performance as that in the c-Si SNSCs.

On the contrary, front-opening crescent SNSCs exhibit significantly improved broadband optical absorption under both TE and TM incidences. Figure 1b, c illustrates that higher *Q*_abs_ can be achieved in the whole band when comparing with the rear-crescent system; the front-opening crescent system greatly outperforms the conventional circular system in a very broad spectral band (300 nm < *λ* < 635 nm), leaving only a narrow band to show the slightly degraded *Q*_abs_. Under the enhanced antenna effect mediated by the front-opening crescent nanostructure, the peaked *Q*_abs_ can be up to 1.97 (1.57) at *λ* = 605 nm (540 nm) under TE (TM) incidence. Ultimately, the front-opening crescent SNSCs exhibit an enhancement of 40.61 % (from 14.38 to 20.22 mA/cm^2^) in *J*_ph_ than that of the conventional circular setup.

**Fig. 2:**
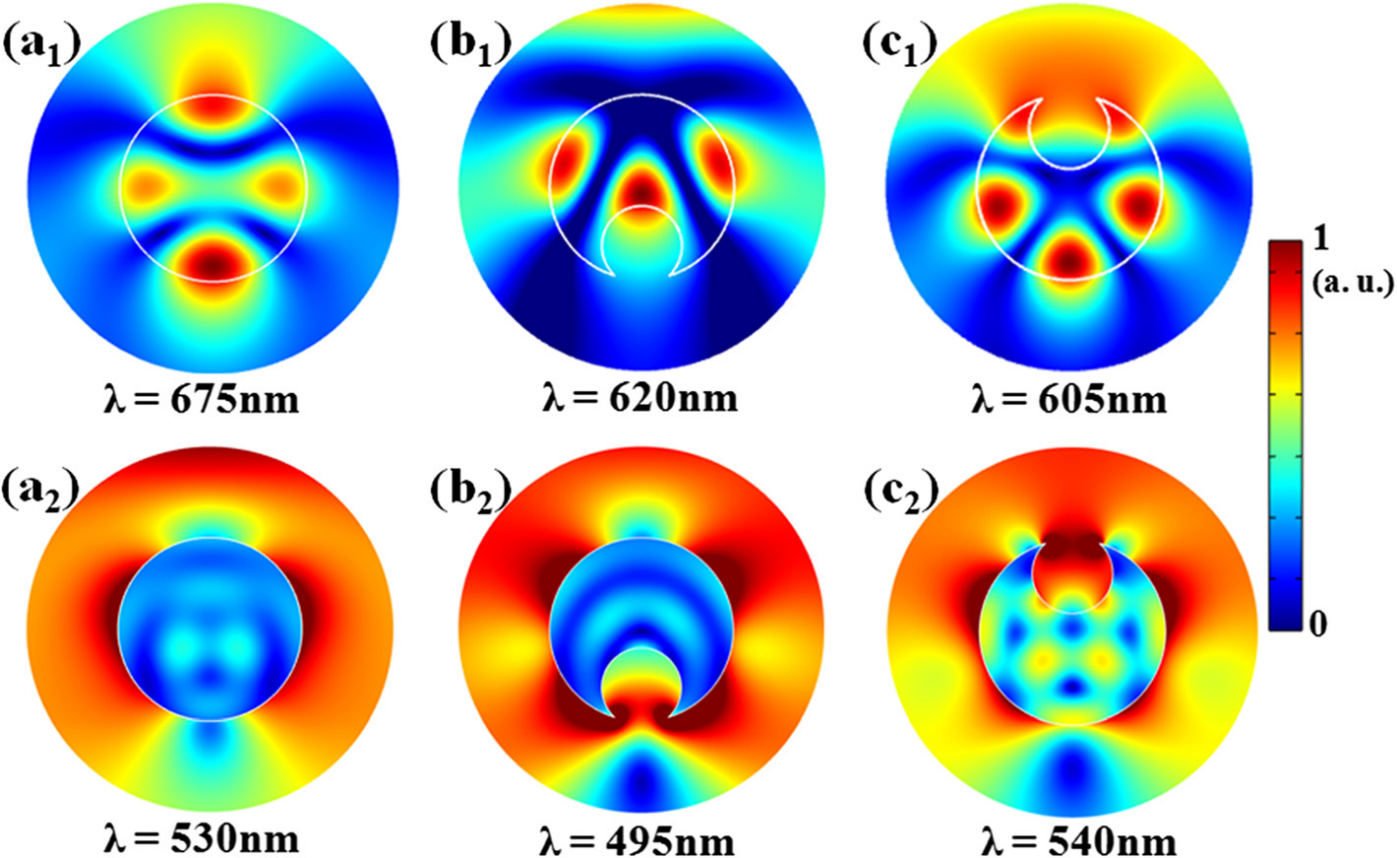
Electric field patterns. Normalized electric field patterns corresponding to the peak wavelengths in Fig. 1b and c for circular (*a*_*1*_)/(*a*_*2*_), rear-opening crescent (*b*_*1*_)/(*b*_*2*_), and front-opening crescent (*c*_*1*_)/(*c*_*2*_) designs under TE/TM incidence

To reveal the physical mechanism behind the outstanding light-trapping performance by the front-opening crescent design, we examine the cross-sectional electric field distribution at several representative wavelengths corresponding to the absorption peaks in Fig. 1b, c. We first focus on the TE cases shown in Fig. 2((a_1_), (b_1_), (c_1_)) for circular, rear-crescent, and front-crescent systems, respectively. For circular NWs, a standard whispering-gallery leaky mode is excited. Modifying the NW cross section into the rear-opening crescent, the resonant characteristics are affected significantly to show stronger peaks but with less number of hot spots (i.e., lower response order), leading to a slightly lower *Q*_abs_ as indicated in Fig. 1b. Nevertheless, the front-opening crescent modulation to the NW cross-section leads to substantially enhanced absorption performance in both the increased number of field hot spots as well as the increased field intensity. The physical reason lies in that the continuously varying crescent tips allows a nearly perfect impedance match between air and the cell, greatly suppressing the reflection and strengthening the optical antenna effect. Therefore, the high performance of the front-opening crescent a-Si:H NW is from both the impedance match as well as the strengthened cavity resonance. However, under TM incidence, the situation changes significantly. This is because the TM illumination has the electric field along the radial direction, along which the NW cavity is too small to support sufficient cavity resonances. Therefore, we could not see the distinct patterns in Fig. 2((a_2_), (b_2_), (c_2_)). In this case, the impedance match plays an important role in coupling the incident light into the NW cavity; therefore, the front-opening crescent configuration has the born benefit in enhancing the absorption performance of SNSCs as displayed in Fig. 2c_2_.

**Fig. 3:**
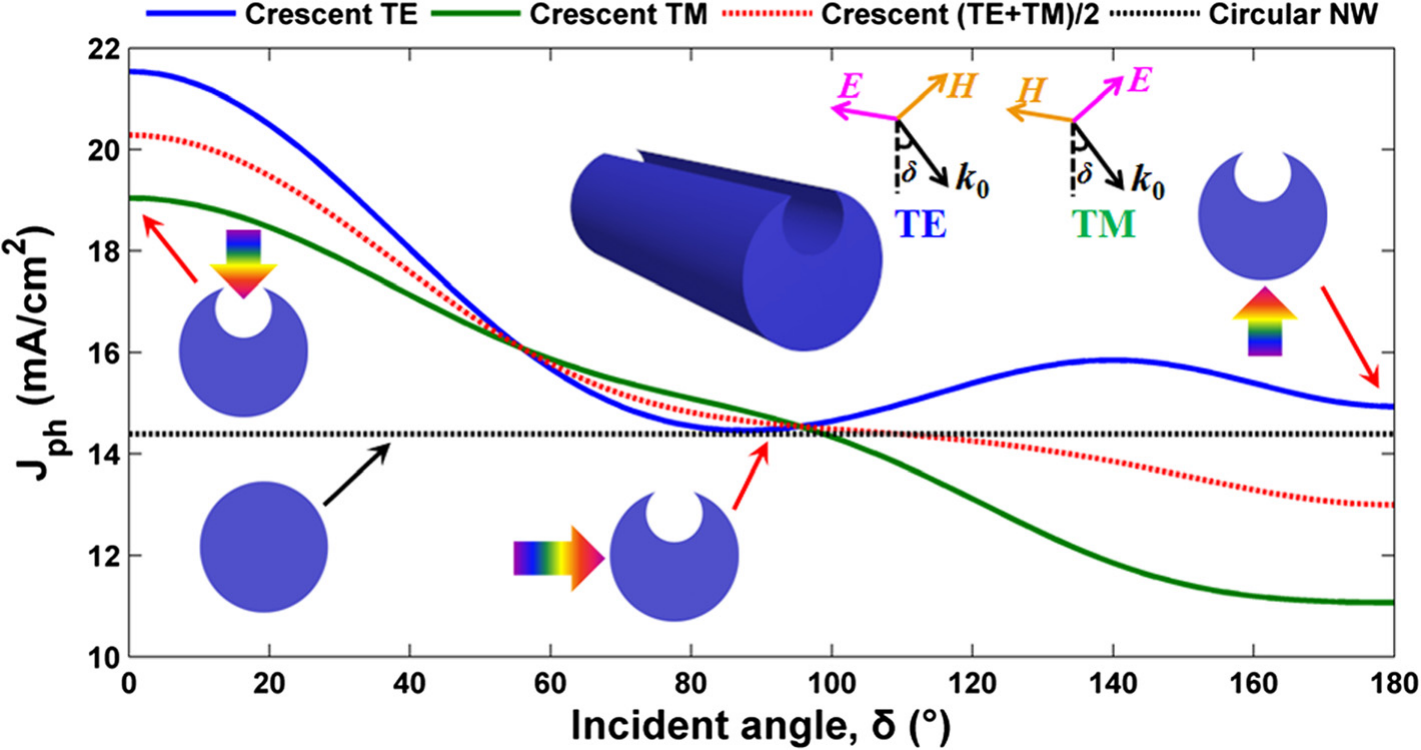
Photocurrent densities. Photocurrent density (*J*_ph_) versus incident angle (*δ*) for the circular and optimized front-opening crescent designs. The *insets* are the schematic diagrams under various cell configurations

Figure 3 plots *J*_ph_ versus *δ* (incident angle) for the optimized crescent NWs with *D* = 200 nm under TE, TM, and unpolarized incidences, respectively, where the circular system is taken to be the reference. Compared to the angle-independent circular system with *J*_ph_ = 14.38 mA/cm^2^, performance is monotonously decreased in front-opening crescent a-Si:H SNSCs with increasing *δ* from 0° to 180° under TM incidence. Under TE incidence, performance is slightly recovered under backward incidence, but the *J*_ph_ value is far lower than that with normal incidence. An overall effect for the unpolarized incidence is with increasing *δ*, the system optical response is gradually degraded; however, the front-opening crescent design can always outperform the circular system as long as *δ* < 90°, which is the case for the most PV systems. Therefore, high optical performance can be sustained under a very wide range of the incident angle by employing the front-opening crescent cross-sectional design for SNSCs.

**Fig. 4:**
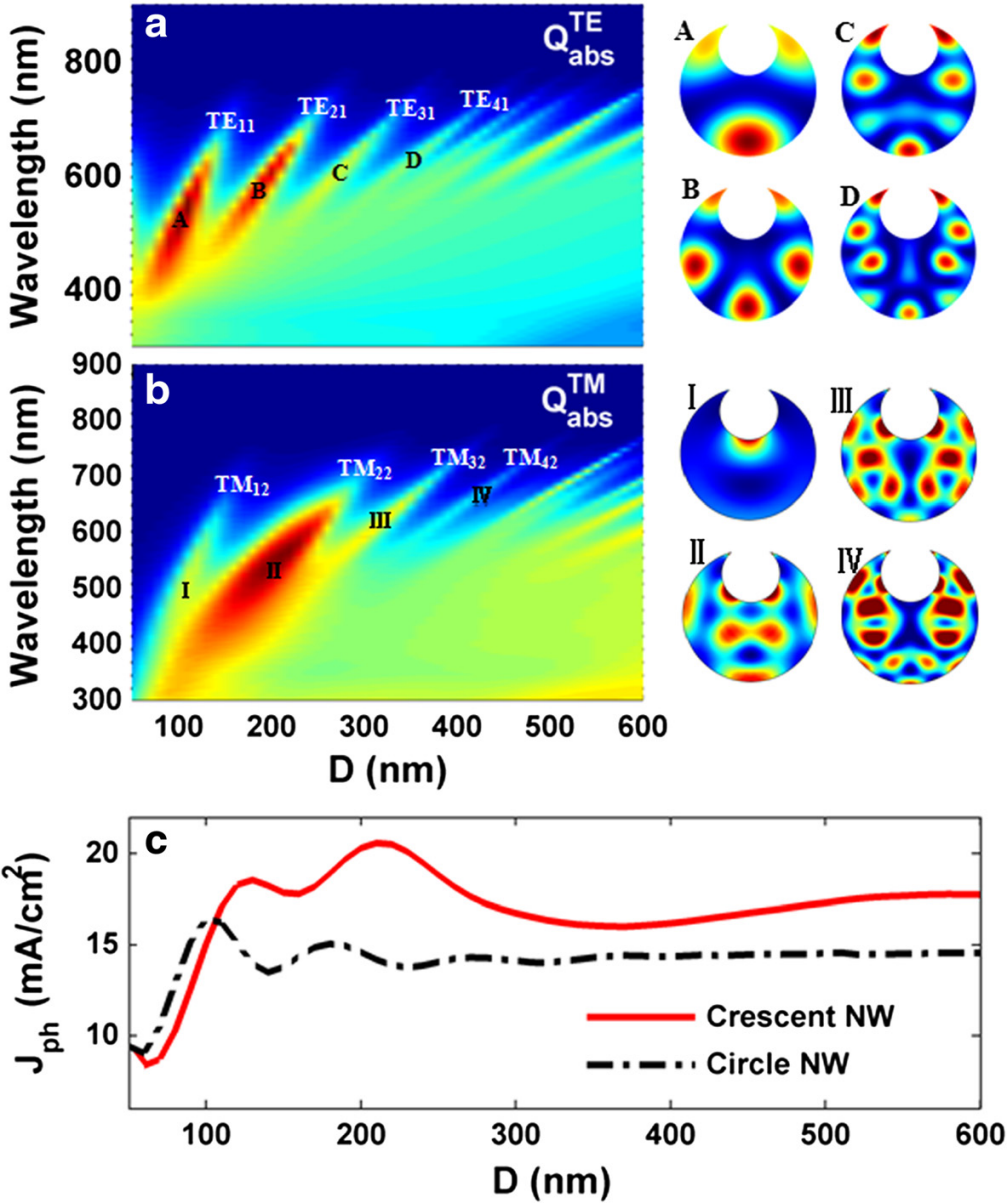
Absorption spectra and electric field patterns. Absorption spectrum (*Q*_abs_) versus *D* under TE (**a**) and TM (**b**) incidences; **c** photocurrent density (*J*_ph_) versus *D*. The electric field patterns of the resonant modes are inserted into the figure

We now examine the tunability of the front-opening crescent a-Si:H SNSCs. Figure 4a, b displays the dispersion characteristics under TE and TM incidences, respectively. In these calculations, *t*/*D* = 0.6 and *θ =* 25° are used to guarantee a constant geometrical morphology. With increasing *D*, it is observed that (1) more high-order optical cavity modes are excited, with the lower-order modes being degenerated/disappearred (the electric field patterns for the resonant modes marked in the figures are displayed on the right side, i.e., patterns A–D and I–IV); (2) the resonant wavelengths are red-shifted distinctly; (3) absorption band is increased but the peaked absorption is decreased; and (4) the strong absorption appears when *D* is relatively small, i.e., the lower-order modes contribute high absorption. Therefore, the strong sensitivity of the absorption performance on the NW size facilitates the control and optimization of the optical response. Figure 4c displays the optimized *J*_ph_ versus *D* for circular and crescent SNSCs under the unpolarized incidence. It is seen that with *D* <100 nm, the circular cell is relatively more capable in light-trapping than the crescent one; however, with increasing *D*, the front-opening crescent system shows a much higher *J*_ph_, especially when 150 nm < *D* < 250 nm.

**Fig. 5:**
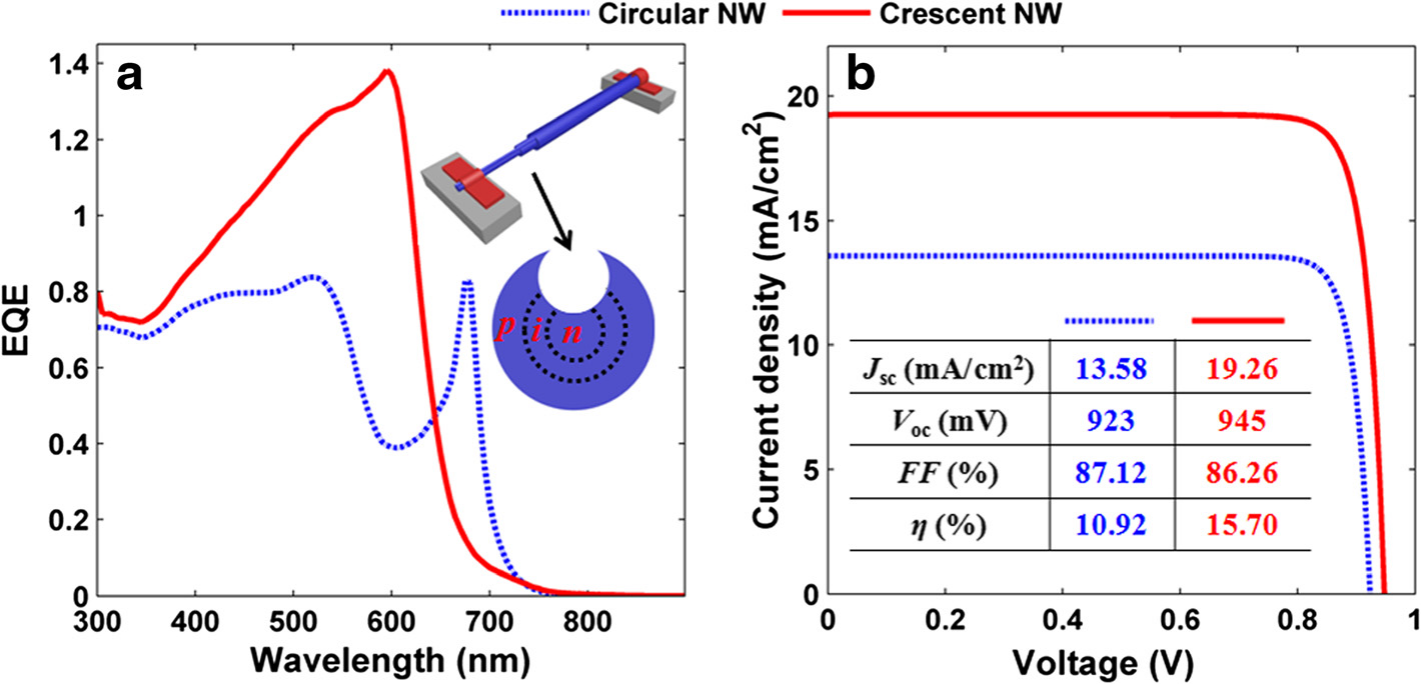
EQE spectra and *I*-*V* characteristics. **a** EQE spectra and (**b**) *I*-*V* characteristics of the front-opening crescent a-Si:H SNSCs with *D* = 200 nm. The device schematic and the tabulated electrical parameters are inserted in (**a**) and (**b**), respectively

In the above optical simulation, we assumed a perfect internal quantum efficiency (i.e., IQE = 100 %), which neglects all kinds of recombination in carrier transportation progress inside the solar cells. To have a comprehensive and actual evaluation on the a-Si:H SNSCs, we perform a detailed electrical simulation [26, 31–35]. The classical electrical parameters of a-Si:H are used in this model, e.g., the doping concentrations of *n* (*p*) regions are 1.3 × 10^18^ (1.3 × 10^17^) cm^−3^; the thicknesses of *p*/*i*/*n* regions are 40/20/40 nm. The rest parameters including intrinsic carrier concentration, carrier mobility, lifetime, and recombination coefficients can be found in reference [35]. Furthermore, two electrodes that are shown in the inserted device schematic in Fig. 5a are employed as perfect ohmic contacts. In this study, we neglect the effect of the collector contacts since the NW length is far beyond than its diameter, leaving a very small portion covered by the electrodes.

Figure 5a shows the simulated EQE spectra of the optimized front-opening crescent and circular a-Si:H SNSCs under an unpolarized incidence. Apparently, realistic carrier transportation process would bring recombination loss for incidences under all wavelengths, leading EQE to be slightly lower than that of *Q*_abs_. As a result, photocurrent density (see the inserted table in Fig. 5b) is reduced from *J*_ph_ = 20.22 (14.38) mA/cm^2^, i.e., integration of *Q*_abs_ under AM1.5G, to *J*_sc_ = 19.26 (13.58) mA/cm^2^, i.e., integration of EQE under AM1.5G, for crescent(circular) a-Si:H SNSCs. Figure 5b plots the current-voltage (*J*-*V*) curves of these two types of a-Si:H SNSCs. Apart from *J*_sc_, the proposed device exhibits a slightly increased open-circuit voltage *V*_oc_ (from 923 to 945 mV), a similar fill factor (FF) (i.e., 87.12 and 86.26 %), and a significantly increased light-conversion efficiency (*η*) from 10.92 to 15.70 % (enhancement ratio is 43.77 %), compared to traditional circular system.

**Fig. 6:**
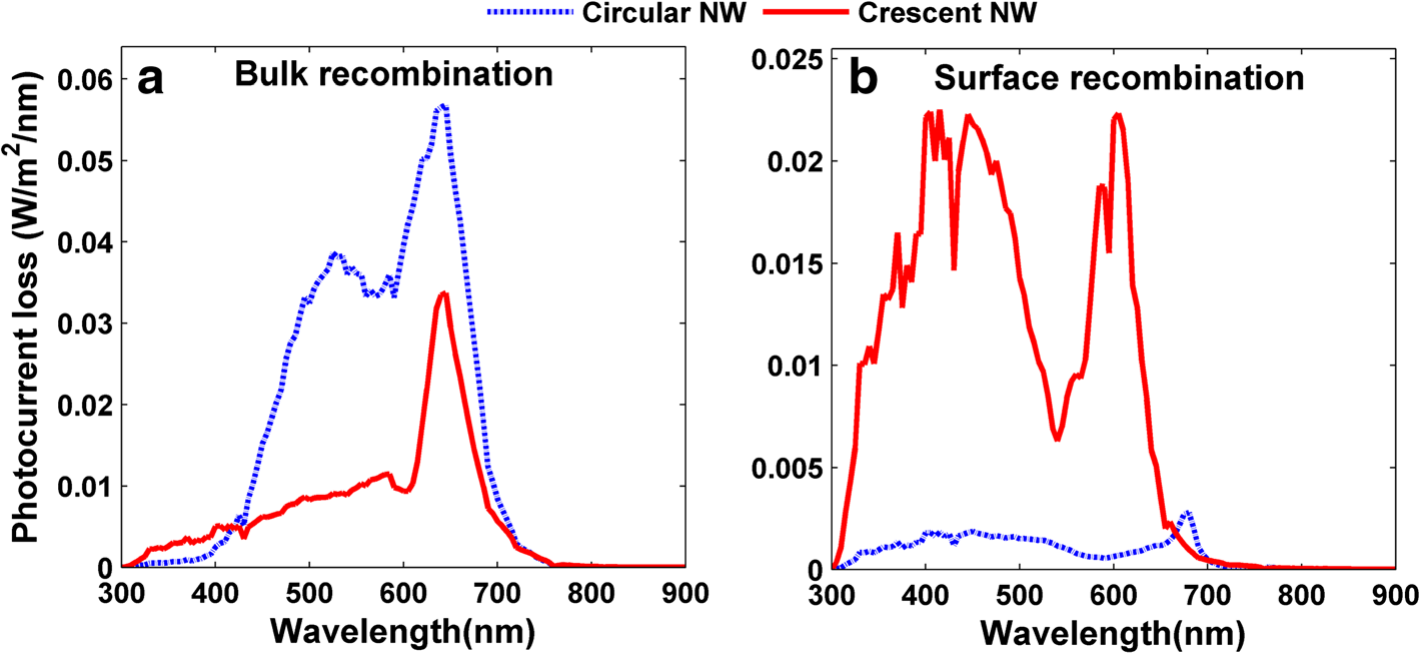
Photocurrent density losses by carrier recombinations. Current density losses arisen from (**a**) bulk and (**b**) surface recombinations for the circular and optimized front-opening crescent designs, respectively

Finally, it is necessary to examine the detailed electronic response of the SNSCs under crescent design since the device has an increased surface area which might lead to a higher surface recombination. Our study indicates that the detailed simulation of the intrinsic carrier transport/recombination processes enables a more accurate prediction on the photocurrent of the device, i.e., the photocurrent is 0.8 (0.96) mA/cm^2^ lower than that based on the purely optical estimation (assuming the lossless internal quantum process) for circular (crescent) SNSCs. The crescent SNSCs exhibits a relatively larger photocurrent loss, revealing a higher carrier recombination. To further explore the recombination processes, Fig. 6a, b quantifies the photocurrent losses due to the bulk and surface recombinations, respectively. It is obvious that the circular SNSCs have a stronger bulk recombination due to the larger device volume; however, the crescent SNSCs exhibit much higher photocurrent loss by surface recombination due to the increased surface area. The combined effect by bulk and surface recombinations results in comparable overall photocurrent losses (i.e., 0.8 and 0.96 mA/cm^2^) by these two kinds of recombination. Therefore, despite that the cell surface is increased by crescent design, the device electrical performance can be well sustained. Moreover, we would like to indicate that the ideally poignant crescent tips have been used in the previous simulations. To take account of the manufacturing capabilities, the blunt tips have to be considered [27]. In our simulations, we have examined the effect of the blunt tips on the absorption performance. It is found that the blunt tips do not qualitatively impact the value of *J*_ph_, i.e., under a representative blunt configuration, *J*_ph_ under TE (TM) incidence is 21.18 (19.10) mA/cm^2^, which is just slightly lower than that of the poignant case, i.e., 21.48 (18.96) mA/cm^2^, showing an average decrement less than 0.40 %.

## Conclusions

In summary, we proposed a design of a-Si:H-based SNSCs with front-crescent cross-sectional morphology in order to obtain broadband and strong optical absorption for efficient photoconversion. By electromagnetic and carrier-transport calculation, we comprehensively evaluated the optoelectronic performance of the crescent a-Si:H SNSCs. Comparisons of front-opening crescent, rear-opening crescent, and conventional circular a-Si:H SNSCs were given under TE, TM, and unpolarized incidences. It was found that the photocurrent density of the front-opening crescent SNSCs can be improved by over 40 % relative to the conventional circular design due to the improved impedance-matching condition as well as the enhanced optical antenna effect. This design has a good tolerance against the change of the incident angle and a high tunability on the optical resonance. Electrical simulation forecasted that the light-conversion efficiency can be up to 15.70 %, showing an enhancement ratio of 43.77 % relative to the circular counterparts.

## Abbreviations

EQEs: external quantum efficiencies
FEM: Finite-element method
FF: fill factor
IQE: internal quantum efficiency
*J*_ph_: photocurrent density
*J*_sc_: short-circuit current density
*J*-*V*: current-voltage
NWs: nanowires
SNSCs: single-nanowire solar cells
TE: transverse electric
TM: transverse magnetic
*V*_oc_: open-circuit voltage
